# Targeted Deletion of Btg1 and Btg2 Results in Homeotic Transformation of the Axial Skeleton

**DOI:** 10.1371/journal.pone.0131481

**Published:** 2015-07-28

**Authors:** Esther Tijchon, Dorette van Ingen Schenau, Fred van Opzeeland, Felice Tirone, Peter M. Hoogerbrugge, Frank N. Van Leeuwen, Blanca Scheijen

**Affiliations:** 1 Laboratory of Pediatric Oncology, Radboud university medical center, Nijmegen, The Netherlands; 2 Laboratory of Pediatric Infectious Diseases, Radboud university medical center, Nijmegen, The Netherlands; 3 Institute of Cell Biology and Neurobiology, National Research Council, Fondazione Santa Lucia 00143, Rome, Italy; 4 Princess Maxima Center for Pediatric Oncology, Utrecht, The Netherlands; The Walter and Eliza Hall of Medical Research, AUSTRALIA

## Abstract

Btg1 and Btg2 encode highly homologous proteins that are broadly expressed in different cell lineages, and have been implicated in different types of cancer. Btg1 and Btg2 have been shown to modulate the function of different transcriptional regulators, including Hox and Smad transcription factors. In this study, we examined the *in vivo* role of the mouse *Btg1* and *Btg2* genes in specifying the regional identity of the axial skeleton. Therefore, we examined the phenotype of *Btg1* and *Btg2* single knockout mice, as well as novel generated *Btg1* ^-/-^;*Btg2* ^-/-^ double knockout mice, which were viable, but displayed a non-mendelian inheritance and smaller litter size. We observed both unique and overlapping phenotypes reminiscent of homeotic transformation along the anterior-posterior axis in the single and combined *Btg1* and *Btg2* knockout animals. Both *Btg1* ^-/-^ and *Btg2* ^-/-^ mice displayed partial posterior transformation of the seventh cervical vertebra, which was more pronounced in *Btg1* ^-/-^;*Btg2* ^-/-^ mice, demonstrating that Btg1 and Btg2 act in synergy. Loss of *Btg2*, but not *Btg1*, was sufficient for complete posterior transformation of the thirteenth thoracic vertebra to the first lumbar vertebra. Moreover, *Btg2* ^-/-^ animals displayed complete posterior transformation of the sixth lumbar vertebra to the first sacral vertebra, which was only partially present at a low frequency in *Btg1* ^-/-^ mice. The *Btg1* ^-/-^;*Btg2* ^-/-^ animals showed an even stronger phenotype, with L5 to S1 transformation. Together, these data show that both *Btg1* and *Btg2* are required for normal vertebral patterning of the axial skeleton, but each gene contributes differently in specifying the identity along the anterior-posterior axis of the skeleton.

## Introduction

The vertebrate axial skeleton is comprised of similar structures that extend from anterior to posterior along the body axis: the occipital skull bones, cervical, thoracic, lumbar, sacral and caudal vertebrae. In mice, there are 30 precaudal vertebrae distributed into seven cervical, thirteen thoracic, six lumbar and four sacral vertebrae [1]. Vertebral development involves two phases, an early stage of somite segmentation from the presomitic mesoderm (PSM) and a later stage of somatic patterning and specification [2]. Segmental identity of the axial skeleton is regulated by a variety of signaling mechanisms and requires the local activation of specific transcriptional regulators, known as Hox genes. This gene family comprises 39 highly conserved transcription factors that are organized into four clusters, including *HoxA*, *HoxB*, *HoxC* and *HoxD* [3, 4]. *Hox* genes are expressed in gradients along the anterior-posterior axis of the body [5–7], and as such control the identity of the axial skeleton. Deregulation of *Hox* gene function leads to homeotic transformations, in which one structure acquires the morphological characteristics of an adjacent homologous structure, a phenotype dictated by the cluster of *Hox* genes that is affected [8, 9]. Other transcriptional regulators important for proper control of vertebral identities include the mammalian Trithorax group (TrxG) and Polycomb group (PcG) proteins, which control the expression of *Hox* genes [10, 11]. Moreover, Hox gene expression is regulated by different signaling pathways, including bone morphogenetic protein (BMP), which is required for normal axial skeletal development [12, 13].

The *B cell translocation gene 1 (Btg1)* and *Btg2* belong to the BTG/TOB family of anti-proliferation genes, and their gene products share 74% protein sequence similarity [14, 15]. Btg1 and Btg2 proteins regulate various cellular processes including proliferation, differentiation and apoptosis, while deregulated expression has been observed in various cancers, including B cell malignancies [16–19]. In addition, the *BTG1* gene is frequently affected by monoallelic deletions in pediatric B-cell precursor acute lymphoblastic leukemia (BCP-ALL), while this has not been observed for *BTG2* [18, 20]. On the other hand, both genes are targeted by point mutations in diffuse large B cell lymphomas [21, 22]. Furthermore, both proteins enhance the transcriptional activity of the homeodomain protein HoxB9, whereas Btg2 was shown to associate with receptor regulated SMAD proteins SMAD1 and SMAD8, thereby activating BMP-dependent transcription [23, 24]. Previous studies using *Btg2^-/-^* mice revealed posterior homeotic transformations of axial skeleton vertebrae, which has been attributed to impaired BMP/Smad signaling [23]. However, it remains to be established whether Btg1 regulates patterning of axial vertebrae and displays similar functions as Btg2.

Several classes of leukemia-associated genes, including Hox transcription factors and their upstream regulator Bmi1, play a critical role in regional patterning of the vertebrate body plan [8, 25–27]. Here, we examined the *in vivo* role of Btg1 and Btg2 in specifying the regional identity of vertebrae along the anterior-posterior axis of the skeleton using both single and double knockout mice for *Btg1* and *Btg2*. This analysis revealed that both Btg1 and Btg2 regulate vertebra specification at the cervical-thoracic and lumbar-sacral junction. On the other hand, Btg2 fulfills a unique role in patterning of the thoracic-lumbar junction, which is not affected in the *Btg1^-/-^* mice.

## Methods

### Experimental Animals

C57BL/6J *Btg1^-/-^* and *Btg2^-/-^* mice have been described earlier [28] and were a kind gift of J.P. Rouault and F. Tirone respectively. [23]. *Btg1^-/-^;Btg2^-/-^* mice were obtained by multiple intercrossing of *Btg1^-/-^* with *Btg2^-/-^* mice. Animals were maintained under specific pathogen-free conditions at our animal facility. All animal experiments were approved by the Animal Experimental Committee of the Radboud university medical center and were performed in accordance with institutional and national guidelines.

Genotyping of mice was routinely performed by PCR, using DNA derived from ear clips. To identify mice carrying either null or wild-type alleles, two primers complementary to the targeted exon 1 (*mBtg1*_F 5’-CCATGCATCCCTTCTACACCC-3’; *mBtg1*_R 5’- TGCAGGCTCTGGCTGAAAGT-3’) and one primer complementary to the neomycin cassette (*mBtg1* Neo_R 5’-CGGAGAACCTGCGTGCAATC-3’) were combined. The wild-type (WT) non-targeted *Btg1* allele was identified as a 136bp PCR fragment with the exon 1 primers, and the *Btg1^-/-^* allele as a 360bp PCR fragment with neomycin specific primer. *Btg2* null wild-type alleles were identified by PCR using four primers, two complementary to the targeted exon 2 for detection of the endogenous *Btg2* allele (*mBtg2*_F 5’- CATCCAAAGGTTCTGGCTATC-3’; *mBtg2*_R 5’- GCCATCACATAGTTCTTCGAG-3’), one complementary to the neomycin cassette and one specific for exon 1 (*mBtg2* Neo_F 5’-CTTCTATCGCCTTCTTGACGAG-3’; *mBtg2* ExI_R 5’-CCACGGGAAGAGAACCGACAT-3’), which were combined in the PCR reaction. The wild-type (WT) non-targeted *Btg2* allele was identified as a 289bp PCR fragment with the exon 2 primers, and the *Btg2^-/-^* allele as a 1372bp PCR fragment with the exon 1 and neomycin specific primers.

### Skeletal Staining and Analysis

Embryos of day 18.5dpc were eviscerated and fixed in 95% EtOH at 4°C. The skeleton was stained for cartilage in 70% ethanol/5% acetic acid and 5% Alcian Blue (8GX, Sigma Aldrich) (0.4% Alcian Blue in 70% EtOH) and bone by addition of 0.005% Alizarin Red S (Sigma Aldrich) for 24–48 hours at room temperature. Embryos were destained in series of gradual lower concentrations of KOH and increasing concentrations of glycerol, and finally stored in 100% glycerol.

The skeletons were scored for axial transformations by counting the number of sternebrae, vertebrae (cervival, thoracic, lumbar and sarcral) and ribs by microscopic analysis.

## Results

### Generation of *Btg1* and *Btg2* knockout mice

**Fig 1:**
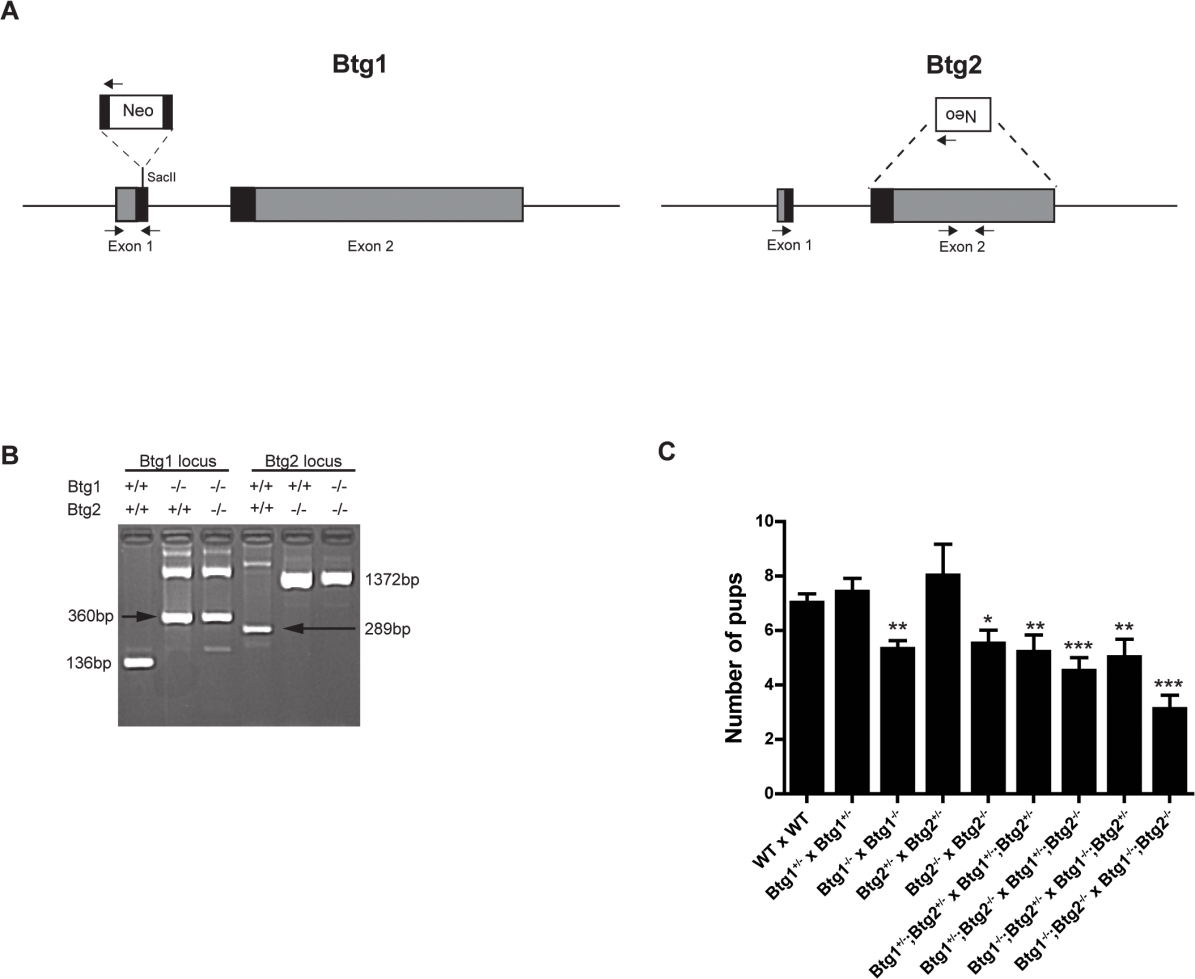
Characterization of *Btg1^-/-^*, *Btg2^-/-^* and *Btg1^-/-^;Btg2^-/-^* mice. (A) The mouse *Btg1* gene is disrupted by insertion of a neomycin resistance cassette via *SacII* restriction sites in the first exon. The second exon of the *Btg2* gene is replaced by a neomycin cassette in the antisense direction. The arrows indicate the position of primers used for genotyping. (B) Genotyping of mice was performed by PCR on genomic DNA using primers specific for the Btg1 and Btg2 wild-type (WT) and knockout (KO) allele. (C) Number of pups obtained from wild-type, heterozygous and homozygous *Btg1*, *Btg2* and *Btg1xBtg2* breedings. Data are from at least 4 independent crossings. *, *P*< 0.05, **, *P*< 0.01, ***, *P*< 0.001.

To investigate the function of Btg1 and Btg2 during normal development we obtained *Btg1^-/-^* and *Btg2^-/-^* single knockout [23, 28], and generated *Btg1^-/-^;Btg2^-/-^* double knockout mice on a *C57Bl/6J* background and examined their appearance for gross abnormalities (Fig 1A and 1B). Genotyping the offspring showed a non-mendelian inheritance pattern for the *Btg1* knockout allele in the single knockout crosses (Table 1), while the *Btg2* knockout allele was strongly underrepresented in the compound crosses (Table 2). The different homozygous knockout animals showed no obvious developmental defects, although the litter size upon interbreeding of the *Btg1*, *Btg2* or *Btg1;Btg2* homozygous knockout lines appeared to be smaller compared to WT animals (Fig 1C).

**Table 1:** Mendelian inheritance in the offspring of *Btg1^-^*, and *Btg2* single knockout mice.

Breeding pairs	% WT exp./obs.	% +/- exp./obs.	% -/- exp./obs.	P-value
WT x WT	100 / 100	0 / 0	0 / 0	n.s (n = 176)
*Btg1^+/+^ x Btg1^+/-^*	50 / 71	50 / 29	0 / 0	<0.0001 (n = 24)
*Btg1^+/-^ x Btg1^+/-^*	25 / 32	50 / 59	25 / 9	0.001 (n = 104)
*Btg1^+/-^ x Btg1^-/-^*	0 / 0	50 / 56	50 / 44	n.s (n = 126)
*Btg1^-/-^ x Btg1^-/-^*	0 / 0	0 / 0	100 / 100	n.s (n = 234)
*Btg2^+/+^ x Btg2^+/-^*	50 / 49	50 / 51	0 / 0	n.s (n = 61)
*Btg2^+/-^ x Btg2^+/-^*	25 / 30	50 / 54	25 / 16	n.s (n = 32)
*Btg2^+/-^ x Btg2^-/-^*	0 / 0	50 / 49	50 / 51	n.s (n = 41)
*Btg2^-/-^ x Btg2^-/-^*	0 / 0	0 / 0	100 / 100	n.s. (n = 177)

Expected (exp.) mendelian inheritance and observed (obs.) inheritance after WT, *Btg1* and *Btg2* breedings.

P-values of the mendelian ratios are calculated with the Chi-square test and are either significant if p <0.05 or not significant (n.s).

**Table 2:** Mendelian inheritance in the offspring of *Btg1; Btg2* double knockout mice.

Breeding pairs	% +/+;+/+ exp./obs.	% +/+;+/- exp./obs.	% +/-;+/+ exp./obs.	% +/-;+/- exp./obs.	% +/+;-/- exp./obs.	% +/-;-/- exp./obs.	% -/-;+/+ exp./obs.	% -/-;+/- exp./obs.	% -/-;-/- exp./obs.	P-value
*Btg1^+/-^;Btg2^+/-^ x Btg1^+/-^;Btg2^+/-^*	12,5 / 6	12,5 / 19	12,5 / 14	12,5 / 22	6,25 / 11	12,5 / 10	12,5 / 5	12,5 / 6	6,25 / 1	<0.0001 (n = 118)
*Btg1^+/-^;Btg2^-/-^ x Btg1^+/-^;Btg2^-/-^*	0 / 0	0 / 0	0 / 0	0 / 0	25 / 25	50 / 60	0 / 0	0 / 0	25 / 15	0.05 (n = 119)
*Btg1^-/-^;Btg2^+/-^ x Btg1^-/-^;Btg2^+/-^*	0 / 0	0 / 0	0 / 0	0 / 0	0 / 0	0 / 0	25 / 56	50 / 44	25 / 0	<0.0001 (n = 40)
*Btg1^-/-^;Btg2^-/-^ x Btg1^-/-^;Btg2^-/-^*	0 / 0	0 / 0	0 / 0	0 / 0	0 / 0	0 / 0	0 / 0	0 / 0	100 / 100	n.s.(n = 71)

Expected (exp.) mendelian inheritance and observed (obs.) inheritance after intercrossing compound *Btg1;Btg2* knockout animals.

P-values of the mendelian ratios are calculated with the Chi-square test and are either significant if p <0.05 or not significant (n.s).

### Homeotic transformation of the axial skeleton in the cervical and thoracic region of *Btg1*- and *Btg2*-deficient mice

To establish whether Btg1 may display distinct or overlapping functions compared to Btg2 in specifying the regional identity of the axial skeleton [23], we analyzed the skeletal defects of *Btg1* and *Btg2* single knockout, as well as *Btg1*;*Btg2* double knockout embryos at the age of 18.5 days post-coitus (dpc). In total, we examined the skeletons of 18 wild-type, 21 *Btg1^-/-^*, 20 *Btg2^-/-^* and 22 *Btg1^-/-^;Btg2^-/-^* animals, which were obtained from three independent breeding pairs.

**Fig 2:**
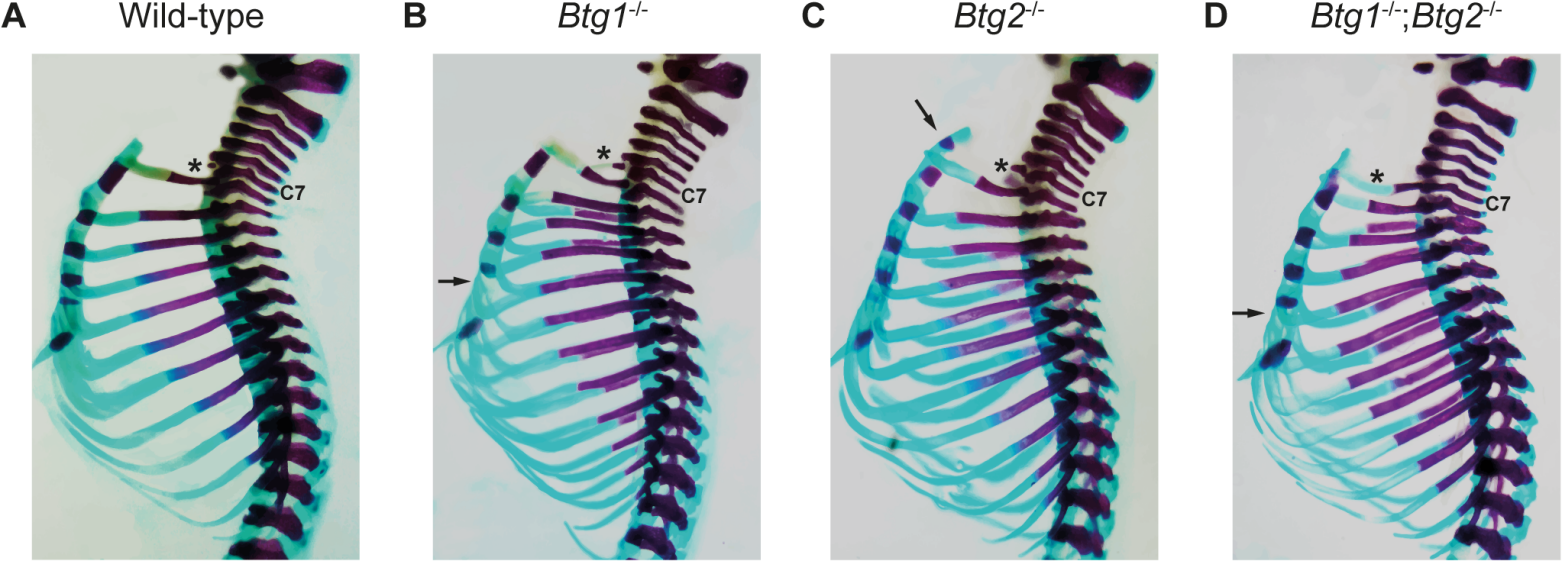
Skeletal abnormalities within the cervical-thoracic region in mice deficient for *Btg1* and *Btg2*. (A-D) Lateral view of the skeletons from 18.5 dpc wild-type, *Btg1^-/-^*, *Btg2^-/-^* and *Btg1^-/-^;Btg2^-/-^* embryos, which were stained with alizarin red and alcian blue. (A-C) Wild-type *C57Bl6/J* mice show a rib anlage at C7 (indicated by asterisk), whereas *Btg1^-/-^* and *Btg2^-/-^* mice display a partial rib at C7 (indicated by asterisk). The extra formed T1 rib in *Btg1* knockout animal is fused to T2 rib. (D) *Btg1^-/-^;Btg2* ^-/-^ double knockout mice display a full rib at C7 that is directly attached to the sternum (indicated by asterisk). (B and D) *Btg1^-/-^* and *Btg1^-/-^;Btg2^-/-^* embryos show often a reduction in the amount of ossified sternebrae (indicated by arrow) compared to wild-type. (C) *Btg2*-deficient mice display occasionally an extra sternebra (indicated by arrow).

Inspection of the upper cervical region in the *Btg1^-/-^*, *Btg2^-/-^* and *Btg1^-/-^;Btg2^-/-^* animals did not uncover any abnormalities in the structure and shape of the atlas (C1) or axis (C2). However, skeletal analysis of the cervical-thoracic junction revealed a common defect among the *Btg1*- and *Btg2*-deficient animals. As expected, most wild-type *C57Bl6/J* mice (72%), showed a small rib anlagen visible as a rudimentary rib attached to the seventh cervical vertebra (C7) (Fig 2A). In contrast, *Btg1*- and *Btg2-*knockout mice displayed a partial or full extensive rib at C7, ranging from 62% in *Btg1^-/-^*, 35% in *Btg2^-/-^* and 95% in *Btg1^-/-^;Btg2^-/-^* mice. As a consequence the presence of the rib anlagen on C7 occurred far less frequently in *Btg1^-/-^* mice (38%) and was almost absent in *Btg1^-/-^;Btg2^-/-^* animals (4.5%) (Table 3). This indicates that in these mice the C7 vertebra acquired the morphological characteristics of the adjacent posterior T1 vertebra, which is therefore termed posterior homeotic transformation. While *Btg1*-deficient mice with a C7 to T1 transformation displayed a fusion of the T1 to the T2 rib without direct attachment to the sternum (Fig 2B), loss of Btg2 resulted in both fusion of the T1 to T2 rib as well as direct attachment of the T1 rib to the sternum (Fig 2C). The *Btg1^-/-^;Btg2^-/-^* double knockout mice displayed a more complete and stronger phenotype, where all mice displayed an extra rib at C7, and in 82% of mice the T1 rib was directly attached to the sternum (Fig 2D).

**Table 3:** Skeletal malformations in *Btg1*- and *Btg2*-deficient mice.

Vertebral transformation	WT(n = 18)	*Btg1^-/-^*(n = 21)	*Btg2^-/-^*(n = 20)	*Btg1^-/-^;Btg2^-/-^*(n = 22)
**Cervical region**				
C7 rudimentary rib	13	8	13	0
C7 → T1 Fused (T1 to T2)	1	13	4	3
Full	0	0	3	18
**Thoracic region**				
Ossified Sternebrae				
#1	18	21	20	22
#2	18	21	20	22
#3	18	21	20	22
#4	18	20	18	22
#5	18	9	14	16
#6	0	0	4	0
Asymmetric sternum	0	1	0	1
Number of Ribs				
12	0	0	14	0
13	18	8	6	22
14	0	13	0	0
Ribs attached to sternum				
6	0	0	0	6
6/7	0	1	1	8
7	18	20	19	8
**Lumbar region**				
T13 → L1 partial	0	0	4	0
complete	0	0	16	21
**Sacral region**				
L5 → S1 partial	0	0	0	5
complete	0	0	0	10
L6 → S1 partial	0	5	0	1
complete	0	1	20	6

**Fig 3:**
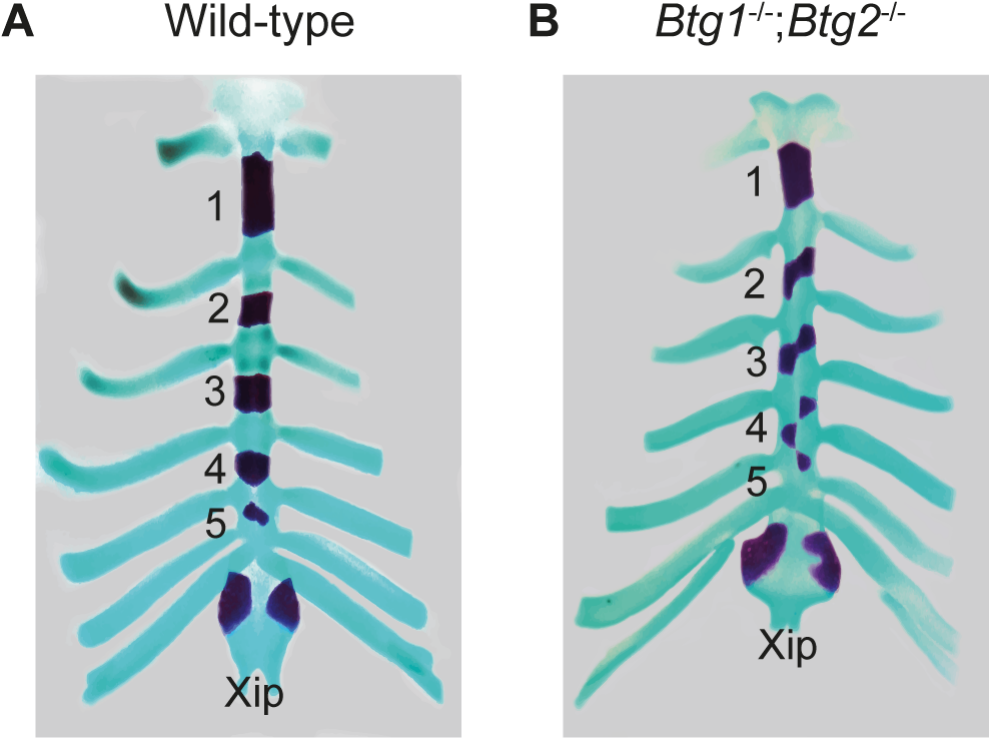
Targeted deletion of *Btg1* and *Btg2* results in malformation of the sternum. (A-B) Ventral view of the sternum with attached ribs of 18.5 dpc wild-type and *Btg1^-/-^;Btg2^-/-^* embryos stained with alizarin red and alcian blue. Wild-type *C57BL6/J* mice display normal ossification of the five sternbrae, whereas an asymmetric pattern of ossification of the sternebrae is observed in mice lacking both *Btg1* and Btg2 expression. The xiphoid process (Xip) of the sternum is not affected in *Btg1-* and *Btg2-*deficient mice.

Normally, the first seven pairs of ribs (T1-T7) form sternocostal junctions with the sternum, where five ossified sternebrae can be distinguished as well as the posterior xiphoid process. In the *Btg1^-/-^* and *Btg2^-/-^* mice we observed that in about 5% of the animals the T7 rib at one side was not attached to the sternum. However, a more severe phenotype was observed in the *Btg1^-/-^;Btg2^-/-^* mice, where 36% of the animals displayed attachment of T7 on only one side and 27% lost the sternocostal junctions of T7 on both sides and had only six ribs attached to the sternum (Table 3). Interestingly, we observed ectopic ossification centers in the sternum of *Btg2*-deficient animals, which resulted in supernumerary sternebrae in 20% of the mice (Fig 2C), probably due to the posterior homeotic transformation. On the other hand, the *Btg1*-deficient mice showed a reduced number of ossified sternebra(e), where the fourth and fifth sternebra lacked mineralization in 62% of mice, which was observed with much lower penetrance in *Btg2^-/-^* (10%) and *Btg1^-/-^;Btg2^-/-^* mice (27%) (Fig 2B–2D). During sternal development, ossification of the cartilage is inhibited at the site where the ribs contact the sternal rudiments. Therefore, this process may become disturbed in the *Btg1^-/-^* mice due to formation of inappropriate connections between the ribs and sternum. In addition, one *Btg1* knockout embryo showed an asymmetric pattern of ossification of the sternebrae, which was even more severe in another mouse lacking both *Btg1* and *Btg2* expression (Fig 3). Presumably, this type of sternal malformation, called crankshaft sternum, results from incorrect positioning of the attachment points of the costal cartilages on either sides of the sternal bar. In conclusion, these data show that both Btg1 and Btg2 regulate specification of cervical and thoracic vertebrae, whereas Btg1 has a dominant function in regulating sternal ossification.

### Btg2 has a unique role in mediating homeotic transformation at the thoracic-lumbar region of the axial skeleton

**Fig 4:**
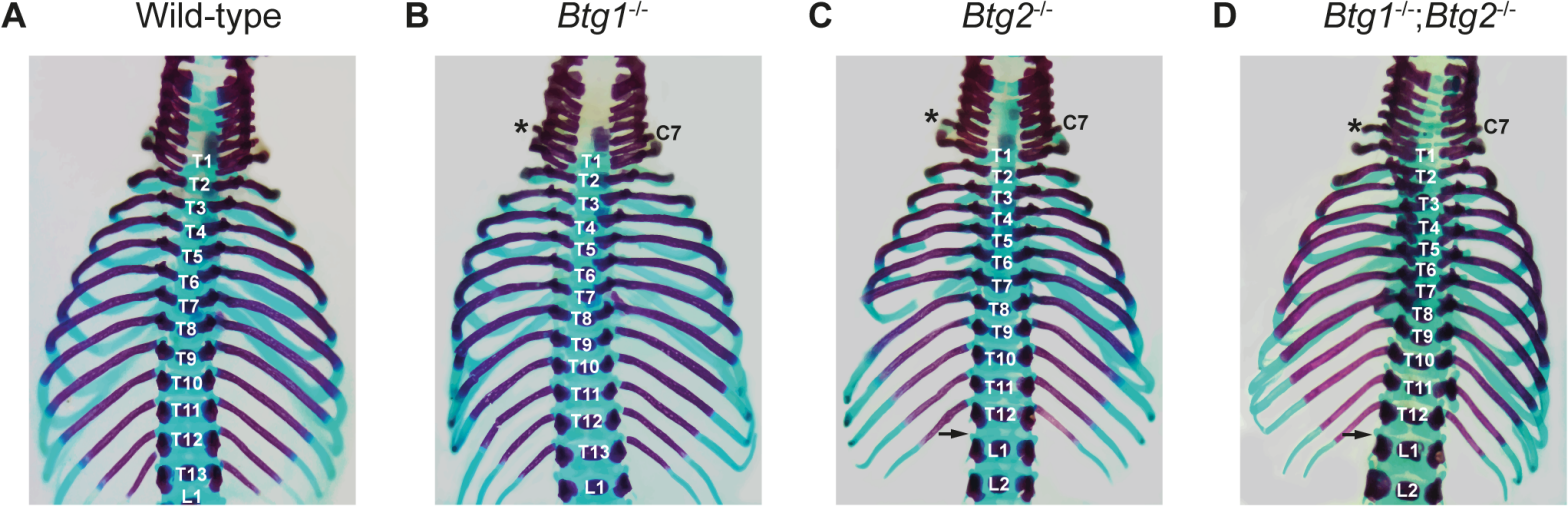
Posterior homeotic transformation of the thirteenth thoracic vertebra in mice deficient for *Btg2*. (A-D) Dorsal view of the cervicothoracic region of the skeleton in 18.5 dpc wild-type, *Btg1^-/-^*, *Btg2^-/-^* and *Btg1^-/-^;Btg2^-/-^* embryos stained with alizarin red and alcian blue. (A-B) Wild-type *C57BL6/J* mice have thirteen thoracic ribs, while *Btg1*-deficient mice display fourteen ribs due to the extra extensive rib at C7. (D) Although the T13 rib is absent in *Btg1^-/-^;Btg2^-/-^* mice they still have thirteen thoracic ribs due to the C7 to T1 posterior transformation.

The rib pairs derived from T8 to T13 are termed “false ribs”, since they do not connect to the sternum. Instead, the T8 to T11 ribs form cartilaginous connections with the adjacent ribs, while T12 and T13 are considered floating ribs, since they form no connections to adjacent rib pairs (Fig 4A). Deletion of *Btg1* resulted in fourteen thoracic ribs in 62% of mice, as a consequence of the extra rib at C7, without any evidence of posterior transformation at the thoracic-lumbar junction (Fig 4B/Table 3). In contrast, mice deficient for *Btg2* displayed thirteen thoracic ribs as they acquired an extra rib at C7, while the thirteenth rib (T13) was often rudimentary or completely absent and acquired the identity of the first lumbar vertebra (L1) (Table 3). As a consequence, most *Btg2^-/-^* mice showed only twelve thoracic ribs compared to thirteen in wild-type animals, and all *Btg2^-/-^* mice displayed posterior homeotic transformation at the thoracic-lumbar junction (Fig 4C). Mice deficient for both *Btg1* and *Btg2* expression showed again normal numbers of thoracic ribs in 95% of the animals, since these mice displayed both C7 to T1 and T13 to L1 posterior transformations (Fig 4D/Table 3). These data demonstrate a unique function for *Btg2* in regulating the regional identity of vertebra at the thoracic to lumbar transition.

### Btg1 and Btg2 function are both required for specifying the vertebral identity at the lumbosacral region

**Fig 5:**
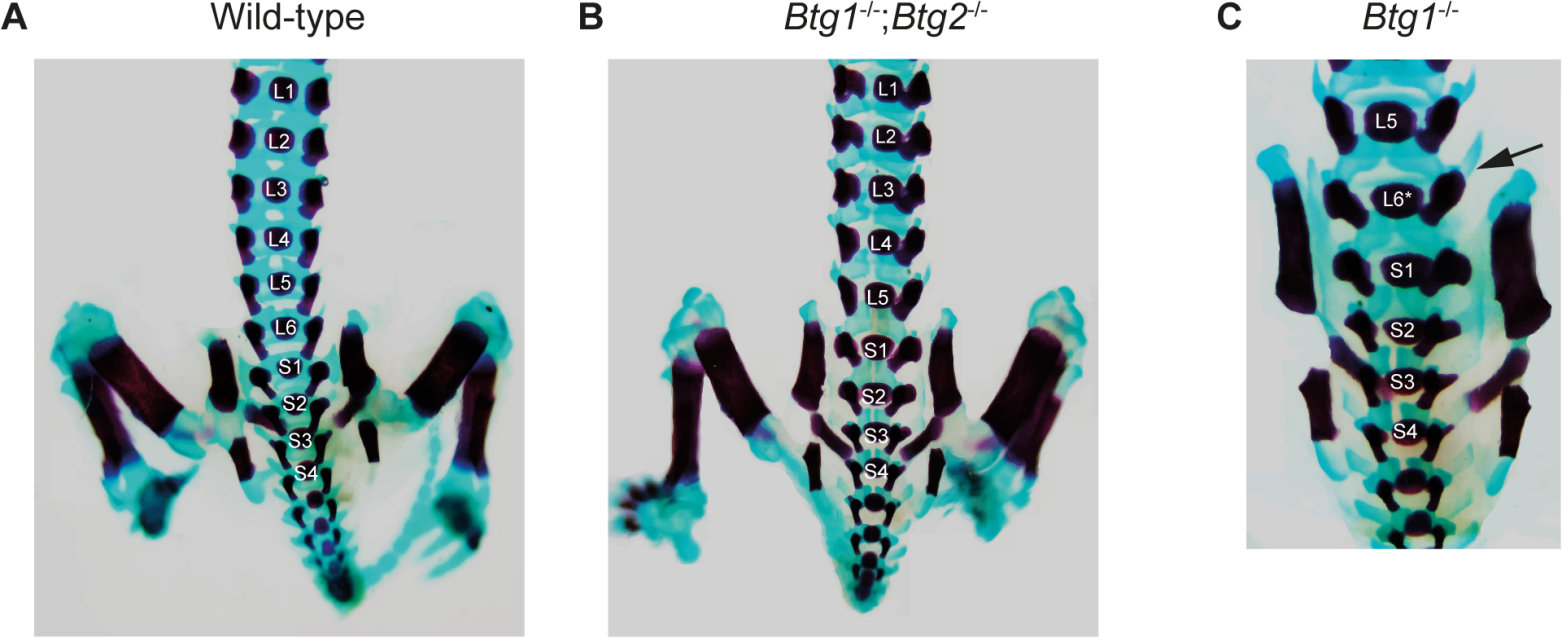
*Btg1;Btg2* double knockout mice display posterior homeotic transformation at the lumbo-sacral transition. Skeletal defects in 18.5 dpc wild-type, *Btg1^-/-^* and *Btg1^-/-^;Btg2^-/-^* embryos. (A-C) Dorsal view showing the lumbar-sacral regions. (B) *Btg1^-/-^;Btg2^-/-^* mice frequently show five lumbar vertebrae compared to six in wild-type mice. (C) *Btg1^-/-^* mice may show asymmetric L6 in which the right side (indicated by arrow) indicates a lumbar vertebra and the left side a sacral vertebra.

Next, we examined the regional identity around the lumbosacral region of the axial skeleton in the *Btg1^-^* and *Btg2*-deleted mice. While *Btg1* single knockout mice showed a partial or complete transformation of the sixth lumbar (L6) vertebra towards the identity of the first sacral vertebra (S1) with a penetrance of 29%, all *Btg2^-/-^* mice displayed a complete transformation of L6 to S1 resulting in 6 lumbar vertebra due to the T13 to L1 transformation (Table 3). Interestingly, we observed a more severe phenotype in mice lacking both *Btg1* and *Btg2* expression, which showed complete transformation of L6 to S1 in 27% of mice, a partial transformation of L5 to S1 with a penetrance of 23% and a complete L5 to S1 transformation in 45% of the *Btg1^-/-^;Btg2^-/-^* mice (Fig 5A–5C). As a consequence, mice with the L5 to S1 homeotic transformation had a reduction in the total amount of lumbar vertebrae. As a consequence of the L6 to S1 and L5 to S1 transformations, an anterior shift was observed in the position of the hindlimb, which is normally always connected to the position of the first sacral vertebra. Together these results demonstrate that there is a synergistic requirement for both *Btg1* and *Btg2* expression in specifying the correct identity of the lumbar vertebrae.

## Discussion

**Fig 6:**
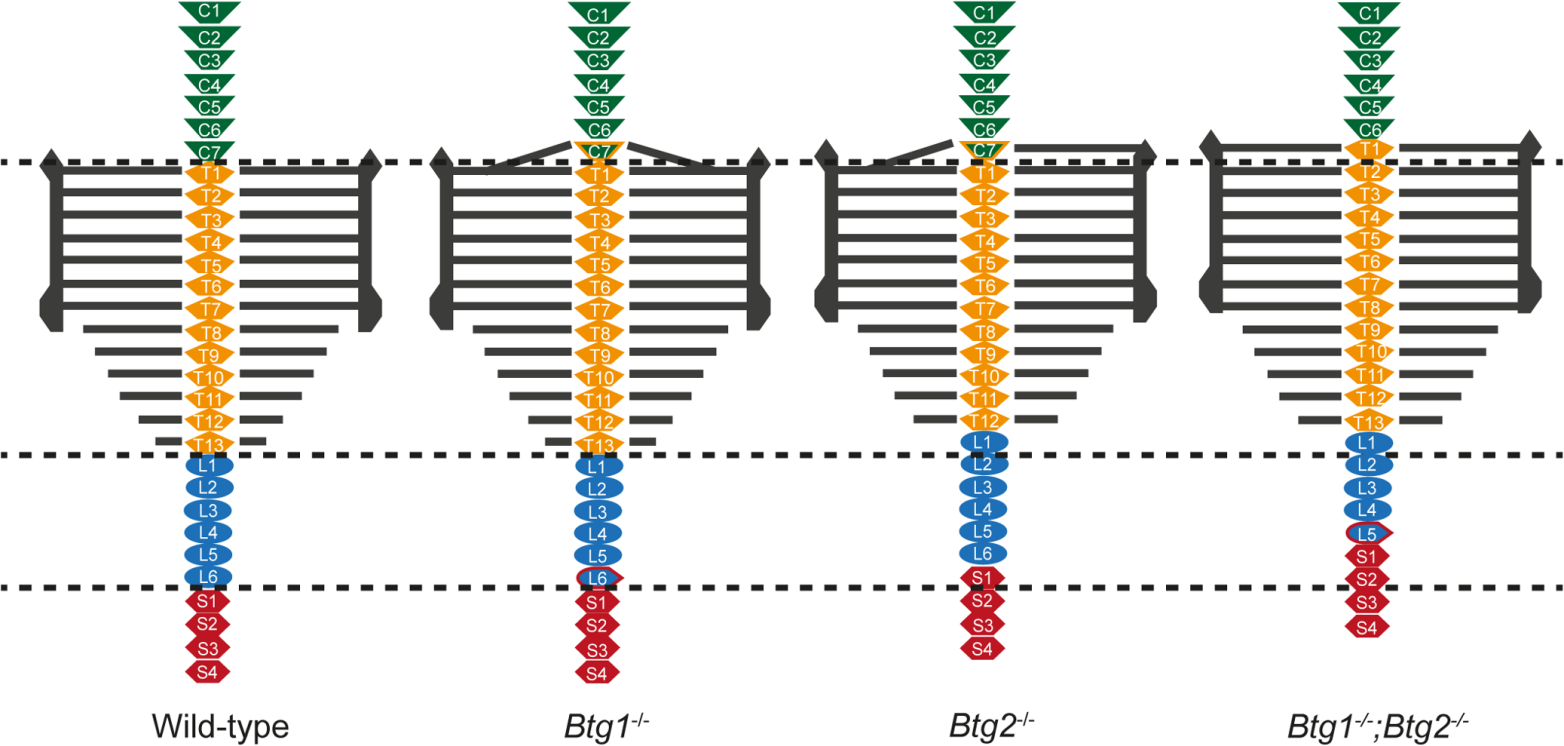
Overview of the skeletal phenotypes in *Btg1-* and *Btg2*-deficient mice. Axial vertebrae are indicated by different colors: green (cervical), yellow (thoracic), blue (lumbar) and red (sacral). The thoracic ribs are indicated by the grey horizontal lines, of which T1-T7 form sternocostal junctions with the sternum. *Btg1-* and *Btg2-*deficient mice display C7 to T1 posterior transformation, while *Btg1* ^-/-^ mice show also partial or complete L6 to S1. *Btg2^-/-^* mice display in most cases T13 to L1 and complete L6 to S1 transformations. *Btg1^-/-^*,*Btg2^-/-^* double knockout animals display a more pronounced phenotype with C7 to T1, T13 to L1 and L6 to S1 homeotic transformations.

The transcriptional cofactors Btg1 and Btg2 represent homologous proteins that regulate cellular proliferation and differentiation in different cell lineages. It was shown previously that *Btg2* is expressed in the presomitic mesoderm (PSM)-tail bud region of the mouse as well as in developing somites and that Btg2 is involved in normal patterning of axial vertebrae [23]. However, a role for Btg1 in regulating the development and regional specification of the mouse skeleton has not been reported so far. In this study, we used both *Btg1*- and *Btg2-*single and double deficient mice, to show that both genes play an essential role in conferring positional information along the anterior-posterior axis of the skeleton. Interbreeding of *Btg1* and *Btg2* homozygous knockout lines resulted in a smaller litter size and a non-mendelian inheritance pattern for the *Btg1* single knockout crosses and the *Btg2* knockout allele in the compound crosses. *Btg1* and *Btg2* are separately implicated in regulating patterning of the lower cervical region, but the combined action of both genes is required for specifying the correct identity of the seventh cervical and the sixth lumbar vertebra. On the other hand, *Btg2* expression appears to be more uniquely involved in the specification of the vertebra at the thoracic-lumbar junction (Fig 6).

Our studies demonstrate that deletion of both *Btg1* and *Btg2* results in an aggravated phenotype, revealing a synergistic effect upon a combined loss of these genes. An extra rib at the seventh cervical vertebra was observed with a low incidence in the *Btg2* knockout mice, and posterior homeotic transformation at this position was more pronounced in the absence of *Btg1* expression. In 95% of the *Btg1^-/-^;Btg2^-/-^* double knockout mice a complete C7 to T1 transformation was observed, arguing that the action of both genes is required for instructing the proper identity of the seventh cervical vertebra. In agreement with previous studies, we found that *Btg2^-/-^* mice displayed homeotic transformation of the thirteenth thoracic vertebra. In contrast, *Btg1*-deficiency had no significant impact on regulating the identity of this last thoracic vertebra, which was also evident from the fact that the double knockout showed a phenotype similar to the *Btg2^-/-^* mice at the thoracic-lumbar junction. Both *Btg1- and Btg2-*deficient mice displayed partial or complete homeotic transformation of the sixth lumbar vertebra towards the first sacral vertebra (L6 to S1), and this phenotype was even more severe in the *Btg1^-/-^;Btg2^-/-^* double knockout mice. Thus, *Btg1* and *Btg2* display both unique and overlapping functions along the anterior-posterior axis in regulating specification of vertebral identity.

Deficiency of *Btg1* resulted in reduced ossification of the distal sternebra(e) as a consequence of delayed ossification within the sternal bands. We also observed abnormal ossification of the sternum where the corresponding costal cartilages invariably inserted into the sternum at different levels at the two sides leading to an asymmetric sternum, known as “crankshaft sternum” [29]. Endochondral bone ossification is regulated by several different signaling pathways, including the action of Runx1 and Runx2 transcription factors [30, 31]. However, it remains to be established whether these defects are primarily the consequence of endochondral ossification defects, or occur secondary to inappropriate connections made between the rib ends and the sternum.

Previously, Btg2 has been considered to regulate skeletal development by modulating BMP/Smad signaling, but the skeletal abnormalities observed in the *Btg1^-^* and *Btg2-*deleted mice are more reminiscent of the phenotype of several knockout mouse models deficient in Polycomb group genes (PcG), including Bmi1, Mel18 and M33 [32–35]. In addition, similar abnormalities have been described for mice deficient in *E2f6* and the spliceosomal protein *Sf3b1*, which are known to associate with a number of PcG proteins. The interaction of PcG proteins with Sf3b1 and E2f6 is essential for the PcG-mediated repression of *Hox* genes [36–40]. Compared to single PcG mutants, the *Bmi1-/-;M33-/-* and *E2f6-/-;Bmi1-/-* double knockout mice reveal extended skeletal transformations [35, 41], due to enhanced deregulation and loss of direct transcriptional control of the *Hox* genes.

Taken together, our data show that *Btg1* and *Btg2* play an important role in anterior-posterior patterning along the vertebral column and both genes fulfill largely overlapping functions in specifying the correct positional identity.
